# Static Balance in Patients with Vestibular Impairments: A Preliminary Study

**DOI:** 10.1155/2016/6539858

**Published:** 2016-06-09

**Authors:** Hossein Talebi, Mohammad Taghi Karimi, Seyed Hamid Reza Abtahi, Niloofar Fereshtenejad

**Affiliations:** ^1^Audiology Department, Musculoskeletal Research Center, Faculty of Rehabilitation, Isfahan University of Medical Sciences, Isfahan 81746-73461, Iran; ^2^Communication Disorders Research Center, Faculty of Rehabilitation, Isfahan University of Medical Sciences, Isfahan 81746-73461, Iran; ^3^Musculoskeletal Research Center, Faculty of Rehabilitation, Isfahan University of Medical Sciences, Isfahan 81746-73461, Iran; ^4^Faculty of Medicine, Isfahan University of Medical Sciences, Isfahan 81746-73461, Iran

## Abstract

*Aims*. Vestibular system is indicated as one of the most important sensors responsible for static and dynamic postural control. In this study, we evaluated static balance in patients with unilateral vestibular impairments.* Materials and Methods*. We compared static balance control using Kistler force plate platform between 10 patients with unilateral vestibular impairments and 20 normal counterparts in the same sex ratio and age limits (50 ± 7). We evaluated excursion and velocity of center of pressure (COP) and path length in anteroposterior (AP) and mediolateral (ML) planes with eyes open and with eyes closed.* Results*. There was no significant difference between COP excursions in ML and AP planes between both groups with eyes open and eyes closed (*p* value > 0.05). In contrast, the difference between velocity and path length of COP in the mentioned planes was significant between both groups with eyes open and eyes closed (*p* value < 0.05).* Conclusions*. The present study showed the static instability and balance of patients with vestibular impairments indicated by the abnormal characteristics of body balance.

## 1. Introduction

Postural control is the ability to control body or posture automatically or in response to external changes [1]. Vestibular, visual, and somatosensory systems are essential and responsible for the control of posture [2]. Afferents of these systems are processed and integrated in multiple structures of central nervous system. Then, motor commands are sent to musculoskeletal system [2]. It is indicated that postural control system plays an important role in maintenance of balance on the small support base provided by the feet [3].

Vestibular system plays an important role in control of static and dynamic posture and balance. Any impairments of movement perception, vertical orientation, control of center of body position, and head fixation result in gait and balance problems [3]. Vestibular pathologies result in balance impairments in 50% of cases [3]. In these conditions, dizziness is one of the most common complaints reported by the patients [3]. Therefore, comprehensive evaluation of the patients with balance disorders is very important.

Instability and postural imbalance are commonly demonstrated as body sway increase in situations with impaired coordination between visual and proprioceptive systems, decrease in threshold of stability and functional capacity, gait changes, and falling in patients with vestibular impairments. Falling (the primary consequence of postural imbalance) is associated with various impairments in neuromuscular system. This condition is resulting from an inability to maintain normal posture [4]. There are some tests that evaluated standing balance in static and dynamic conditions. Berg Balance Scale was a subjective test designed to evaluate standing balance in elderly patients [5]. This scale predicts falling in seniors and is sensitive to change after vestibular rehabilitation [6]. The Get Up and Go Test designed to identify elderly fallers is a test of walking balance. It differentiates elderly patients with high risk of falling from those patients with low risk of falling [7]. Vestibuloocular reflex (VOR) and vestibulospinal reflex (VSR) are considered for the objective assessment of postural stability in patients with vestibular impairments [4]. VOR is indicated as a system of primary control of visual stabilization during locomotion and any disturbances of VOR result in dizziness and other symptoms of body imbalance [8]. Assessment of VOR and VSR is essential for vestibular, visual, and somatosensory systems responsible for balance control [9]. Many studies highlighted more detailed information about the role of VOR in patients with vestibular impairments. In contrast, far more studies are required for assessment of VSR and proprioception in the mentioned patients.

In the present study, we evaluated this reflex and the proprioception in more detail using force plate platform in patients with vestibular impairments. Our primary hypothesis was that this reflex could be affected in the patients with vestibular impairments. The results of this study surely shed light on and increase our knowledge about the effects of vestibular impairments on VSR and proprioception and better designing of balance rehabilitation programs.

It is indicated that the assessment of VSR as well as proprioception has been conducted in patients with vestibular impairments mostly with posturography [10]. In the present study, we measured many components of static balance such as center of pressure (COP) in anteroposterior (AP) and mediolateral (ML) directions, path length (PL), and velocity in the mentioned directions using force plate platform.

## 2. Materials and Methods

10 patients with unilateral vestibular impairments and 20 normal counterparts in the same sex ratio and age limits (50 ± 7) participated in this study. The patients were referred from ear, nose, and throat clinic of Alzahra Hospital of Isfahan University of Medical Sciences. All of the patients showed evidence of vestibular impairments such as true vertigo. These patients were evaluated by an otolaryngologist and an expert audiologist using videonystagmography (VNG) test. All of the patients had at least 20% weakness on bithermal caloric testing in the left ear. The patients also had decreased vestibuloocular reflex responses to low frequency sinusoidal rotations in darkness. All of the patients have been medicated by ENT surgeons.

### 2.1. Inclusion Criteria 

 Inclusion criteria were as follows: presence of vestibular impairments, age limit between 30 and 60 years, no evidence of cerebellar ataxia, no evidence of effusions in the middle ear, normal condition in musculoskeletal system and normal standing and gait, normal visual function, and no evidence of neurologic disorders such as multiple sclerosis and sensory impairments of the lower limbs in the diabetic patients.

### 2.2. Exclusion Criteria 

 Exclusion criteria were as follows: all of the patients with Meniere's disease, migraine, joint replacements, history of neurologic disease, and functional vision with their corrective lenses had been excluded from this study.

The present study was in accordance with Helsinki Declaration. We conducted this project in Musculoskeletal Research Center of Isfahan University of Medical Sciences. An ethical approval was obtained from ethical committee of Isfahan University of Medical Sciences (Ethics Committee reference number IR.MUI.REC.1394.2.094). Moreover, every subject was asked to sign a consent form before data collection. The static balance of the participants while standing was evaluated by use of Kistler force plate (Kistler Instrument Corp., NY, United States) when they were standing for one minute with eyes open and eyes closed. They were asked to stand on force plate for one minute in a comfortable position. The subjects were asked to keep their eyes either open or closed while maintaining the stance (feet together and arms to the sides) for 60 s [11]. The data were collected with frequency of 120 Hz and were filtered with Butterworth low pass filter with cutoff frequency of 10 Hz. The first and last 15 seconds of the data were deleted to remove the effects of sudden standing on the force plate and also the effects of fatigue, respectively. The tests were repeated to collect 5 trials for each condition (eyes open and eyes closed). It should be emphasized that the selection of conditions to be tested was done randomly. The stability parameters for static balance of the participants were evaluated by use of the following equations:

(1) COPEAP mm=Xmax−Xmin,COPEML mm=Ymax−Ymin,PLAP mm=∑n−1xi+1−xi2,PLML mm=∑n−1yi+1−yi2,VAP mm/min=∑n−1xi+1−xi2t,VML mm/min=∑n−1yi+1−yi2t,

where *X* plane is the displacement in the anteroposterior direction and *Y* plane is the displacement in the mediolateral direction. In addition, COPEAP, COPEML, PLAP, PLML, VAP, and VML are the excursion of the center of pressure in the anteroposterior direction, excursion of the center of pressure in the mediolateral direction, path length in the anteroposterior direction, path length in the mediolateral direction, velocity of the COP in the anteroposterior direction, and velocity of the COP in the mediolateral direction, respectively.

### 2.3. Statistical Analysis

We measured the mean of the parameters for 5 test trials. In addition, we compared static balance between patients with vestibular impairments and normal participants with two-sample *t*-test. The significant point at 0.05 was used for final analysis. The statistical test was done using SPSS software (version 21, Chicago, USA).

## 3. Results

Table 1 shows the characteristics of the subjects that participated in this study. The mean values of COPEAP and COPEML in the patients with eyes open were 25.93 ± 9.6 and 18.18 ± 7.04 mm, respectively, compared to 25.25 ± 10.82 and 13.76 ± 5.32 mm for normal subjects (the difference between the mean values between both groups was not significant, *p* value > 0.05).

There was also no significant difference between COPEAP and COPEML between both groups with eyes closed (*p* value > 0.05). In contrast, the difference between VAP and VML between both groups with eyes open and eyes closed was significant (*p* value = 0.00). The PLAP and PLML were 460.77 ± 62.62 and 502.19 ± 77.2 and 1345.75 ± 571.12 and 970.62 ± 310.35 mm, for normal cases and patients, respectively (*p* value = 0.00). Also, with eyes closed, there was significant difference between normal participants and patients for PLAP and PLML (*p* value = 0.01).  Table 2 summarizes the stability parameters of both normal participants and the patients.

## 4. Discussion

There is no doubt that the risk of falling mostly depends on static and dynamic stability of subjects. Various neuromuscular disorders influence the stability of subjects while standing and walking [12–15]. In addition, some patients suffer from vestibular impairments which influence their abilities to manage their stability [11, 15, 16]. The present study compared the stability of the patients with vestibular impairments with that of normal subjects during quiet standing.

The results showed that although it seems that these patients have the same stability as that of normal subjects (based on COP excursions), they have impaired stability. They have the same excursion while standing as normal subjects but they have continued sways. It means that their sway range is the same as normal but they do not have a constant stability. Their COP moved from anterior to posterior position and from lateral to medial position simultaneously with high frequency. In other words, they cannot control the movement of COP. These results support findings reported in some researches that instability was consistently increased in patients with vestibular impairments [11, 15].

This study suggested that unilateral vestibular dysfunction increased postural instability compared to control participants. The patients showed a significant increase approximately in all parameters of static stability. Our results demonstrated that the parameters assessed by force plate platform could be sensitive in showing any alterations in static stability of patients with vestibular impairments. This study highlighted the static instability of patients with vestibular impairments indicated by the abnormal characteristics of body balance. Some studies showed that the kinetics provide a useful third level of analysis for patients with vestibular impairments [17, 18]. These analyses with the other balance measurements including Berg Balance Scale (Berg), Dynamic Gait Index (DGI), Timed Up and Go (TUG), Computerized Dynamic Posturography Sensory Organization Test (SOT), and VNG are sensitive in identifying patients with vestibular impairments [17, 18]. For this preliminary study, our data may not be strictly comparable to the general population. These data should be interpreted with caution, however, due to the decreased sample size. In addition, further research could be suggested to better identify the consequences of vestibular impairments on static and dynamic stability.

## 5. Conclusion

In this research, the measured parameters of static balance including center of pressure, path length, and velocity in anteroposterior and mediolateral directions show static balance abnormality in patients with vestibular impairments.

## Figures and Tables

**Table 1:** The characteristics (mean ± SD) of the subjects that participated in this study.

Participants	Number	Age	Mass	Height (m)	BMI (kg/m^2^)	Sex (female)
Patients	10	50 ± 7	59 ± 14	1.7 ± 0.25	20.50 ± 5.2	5
Normal	20	50 ± 5	61 ± 12.5	1.7 ± 0.15	21.20 ± 4.3	10

**Table 2:** The mean values with standard deviation of the stability parameters of normal subjects and the patients.

Participants		COPEAP (mm)	COPEML (mm)	VAP (mm/min)	VML (mm/min)	PLAP (mm)	PLML (mm)
Eyes open	Normal	25.25 ± 10.82	13.76 ± 5.32	928.22 ± 113.5	1004.45 ± 154.43	460.77 ± 62.62	*502.19 ± 77.2*
	Patients	25.93 ± 9.6	18.18 ± 7.04	2688.83 ± 1139.73	1939.89 ± 621.34	1345.75 ± 571.12	*970.62 ± 310.35*
	*p* *value*	*0.43*	*0.06* ^*∗*^	*0.00* ^*∗*^	*0.00* ^*∗*^	*0.00* ^*∗*^	*0.00* ^*∗*^

Eyes closed	Normal	24.21 ± 9.76	14.55 ± 8.06	985.56 ± 160.49	1005.38 ± 173.27	492.31 ± 80.69	*502.88 ± 86.68*
	Patients	30.55 ± 18.56	19.46 ± 12.00	2470 ± 1023	1974.33 ± 698.81	1235 ± 511.5	*987.14 ± 349.42*
	*p value*	*0.18*	*0.14*	*0.00* ^*∗*^	*0.001* ^*∗*^	*0.001* ^*∗*^	*0.001* ^*∗*^

^*∗*^Significant difference between normal subjects and patients with vestibular impairments at *p* < 0.05.
